# Conversion and Hydrothermal Decomposition of Major Components of Mint Essential Oil by Small-Scale Subcritical Water Treatment

**DOI:** 10.3390/molecules25081953

**Published:** 2020-04-22

**Authors:** Tai-Ying Chiou, Shiori Nomura, Masaaki Konishi, Chien-Sen Liao, Yasutaka Shimotori, Miki Murata, Naofumi Ohtsu, Yoshihito Kohari, Wei-Ju Lee, Tsung-Yu Tsai, Yuichi Nagata, Tohru Saitoh

**Affiliations:** 1 School of Regional Innovation and Social Design Engineering, Kitami Institute of Technology, Koen-cho, Kitami, Hokkaido 090-8507, Japan; konishim@mail.kitami-it.ac.jp (M.K.); yasu@mail.kitami-it.ac.jp (Y.S.); 2 Department of Biotechnology and Environmental Chemistry, Kitami Institute of Technology, Koen-cho, Kitami, Hokkaido 090-8507, Japan; m1852500115@std.kitami-it.ac.jp; 3 Department of Biological Science and Technology, I Shou University, Yanchao District, Kaohsiung 824–45, Taiwan; csliao@isu.edu.tw; 4 School of Earth, Energy and Environmental Engineering, Kitami Institute of Technology, Koen-cho, Kitami, Hokkaido 090–8507, Japan; muratamk@mail.kitami-it.ac.jp (M.M.); nohtsu@mail.kitami-it.ac.jp (N.O.); kohari@mail.kitami-it.ac.jp (Y.K.); saitoh@mail.kitami-it.ac.jp (T.S.); 5 School of Food Safety, Taipei Medical University, Xinyi District, Taipei 110–31, Taiwan; weijulee@tmu.edu.tw; 6 Department of Food Science, Fu Jen Catholic University, Xinzhuang District, New Taipei City 242–05, Taiwan; tytsai@mail.fju.edu.tw; 7 Kitami Hakka Tsusho Co., Ltd., Oroshi-machi, Kitami, Hokkaido 090–0056, Japan; oiwa@hakka.be

**Keywords:** subcritical water treatment, mint essential oil, conversion, hydrothermal decomposition, thymol

## Abstract

Thermal stabilities of four major components (*l*-menthol, *l*-menthone, piperitone, and *l*-menthyl acetate) of Japanese mint essential oil were evaluated via subcritical water treatment. To improve experimental throughput for measuring compound stabilities, a small-scale subcritical water treatment method using ampoule bottles was developed and employed. A mixture of the four major components was treated in subcritical water at 180–240 °C for 5–60 min, and then analyzed by gas chromatography. The results indicated that the order of thermal resistance, from strongest to weakest, was: *l*-menthyl acetate, *l*-menthol, piperitone, and *l*-menthone. In individual treatments of mint flavor components, subsequent conversions of *l*-menthyl acetate to *l*-menthol, *l*-menthol to *l*-menthone, *l*-menthone to piperitone, and piperitone to thymol were observed in individual treatments at 240 °C for 60 min. As the mass balance between piperitone and thymol was low, the hydrothermal decomposition of the components was considered to have occurred intensely during, or after the conversion. These results explained the degradation of mint essential oil components under subcritical water conditions and provided the basis for optimizing the extraction conditions of mint essential oils using subcritical water.

## 1. Introduction

Mint essentials oils are well-known and popular products for their cooling and invigorating effects. Mint essential oils are generally obtained from Japanese mint (*Mentha arvensis L.*), peppermint (*Mentha piperita, L.*), and spearmint (*Mentha spicata L.*). *l*-Menthol is the most abundant component in the Japanese mint and peppermint essential oils, while *l*-carvone is the most abundant component in spearmint essential oil [1,2]. Japanese mint essential oil is renowned for having a higher *l*-menthol content than that of peppermint essential oil, and the proportion of *l*-menthol in Japanese mint essential oil can exceed 70% [3]. Hydrodistillation is the most universal and conventional method for obtaining essential oil from mint [4]. However, the drawback of this method is the time-consuming extraction. For example, hydrodistillation requires three hours for a batch extraction of rosemary essential oil [5]. As a promising alternative, subcritical water treatment was reportedly more efficient than hydrodistillation in the extraction of marjoram essential oil [6]. 

Subcritical water is also known as hot pressured water, its temperature being above the boiling point (100 °C), and below the critical point (374 °C). The liquid state of subcritical water is maintained by an external force or its own high vapor pressure in an enclosed space. As subcritical water has highly ionic characteristics and a low dielectric constant (ε), it is regarded as an excellent solvent for obtaining both hydrophobic and hydrophilic substances from botanical materials [7,8,9,10]. In our recent studies, subcritical water demonstrated remarkable extraction efficiency for recovering total carbohydrate, protein, and phenolic contents from Japanese mint leaves within a 5 min treatment [11]. In the standard batch-wise extraction by subcritical water, mint essential oil is extracted along with dissolved substances, such as carbohydrates and proteins, necessitating additional separation operations for recovering the essential oil. In order to minimize the effort and improve extraction efficiency, we have proposed a modified method, involving subcritical water treatment coupled with pressure-releasing distillation, to obtain essential oil from Japanese mint [12]. By employing this specialized method for the recovery of essential oil, we could avoid the step of separating the liquid from solid residue, and the highest total yield of 30.5 mg/g-dry leaves of essential oil was achieved with a rapid extraction time of 5 min at 180 °C. 

The highly ionic characteristics and a low dielectric constant were regarded as pivotal for improving the extraction efficiency, however, the phenomena of conversion and decomposition of mint essential oil components were observed at higher extraction temperatures and extended extraction times during subcritical water treatment [11,12]. Conversion and decomposition were considered to be the reason for loss of product during extraction. Thus far, materials subjected to subcritical water treatment have been natural products, and analysis of extract compositions has been too complicated. Therefore, the phenomena of conversion and decomposition have not been clarified. In order to further improve the extraction efficiency and reduce the loss of essential oil during subcritical water treatment, it is necessary to understand the mechanisms involved in conversion and decomposition of essential oil components under subcritical water conditions. The aim of this study is to investigate the possible mechanism of conversion and decomposition of the major components of mint essential oil during subcritical water treatment. The four major components, *l*-menthol, *l*-menthone, piperitone, and *l*-menthyl acetate, were subjected to subcritical water treatment, and the batch-wise method was employed. Moreover, in order to improve experimental throughput for measuring compound stabilities in subcritical water treatment, a small-scale technique utilizing ampoules was developed and employed. Since the optimized treatment condition was indicated to be 180 °C for 5 min in our previous study [11,12], the longer treatment time and higher treatment temperature were examined in this study to further evaluate the thermal stability of the major components.

## 2. Results and Discussion

### 2.1. Degradation of the Components by Subcritical Water Treatment

**Figure 1:**
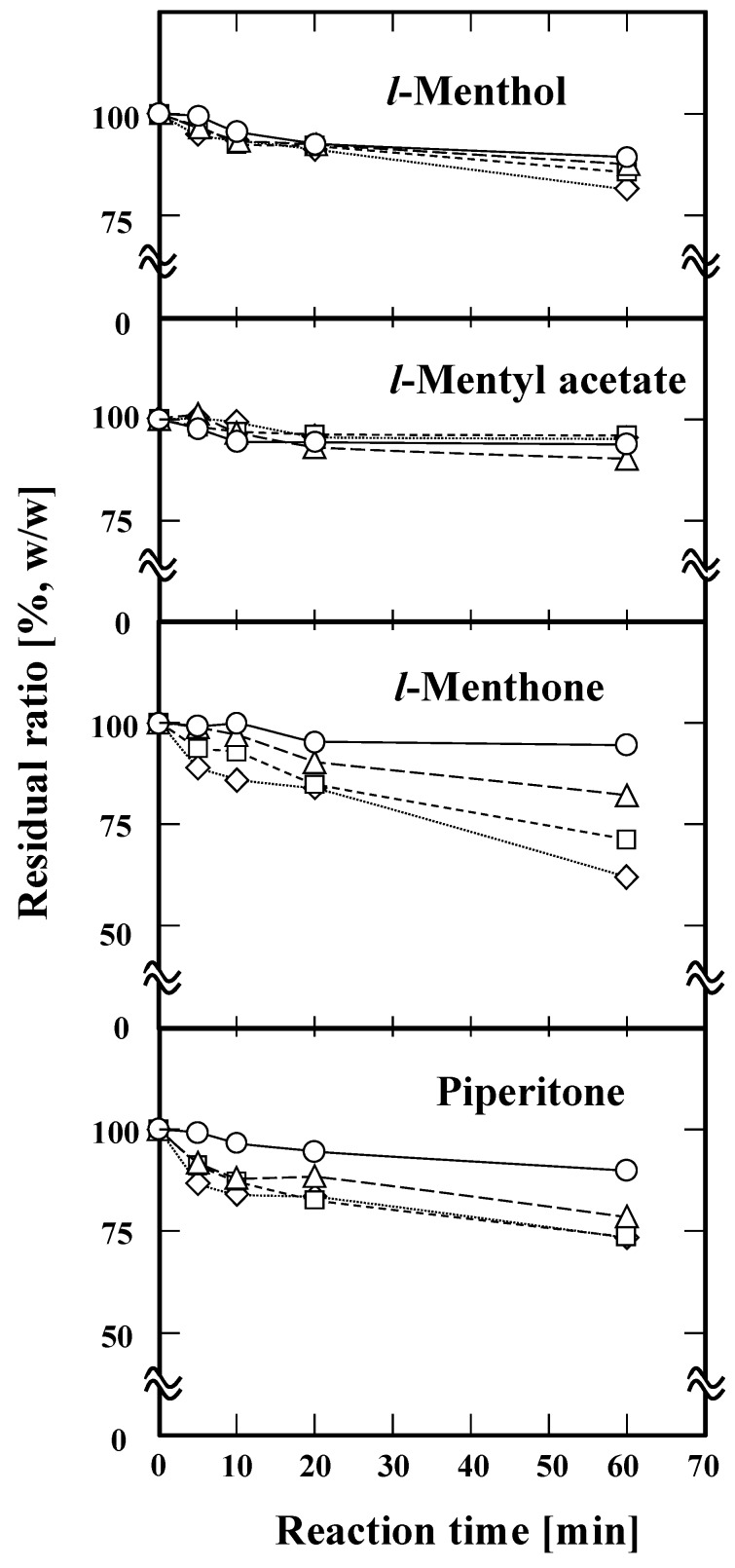
Subcritical water treatments of the mixture containing *l*-menthol, *l*-menthyl acetate, *l*-menthone, and piperitone. The subcritical water treatments were carried out at 180 °C (○), 200 °C (△), 220 °C (□), and 240 °C (◇). The reaction time was carried out for 5, 10, 20, and 60 min, respectively.

Small-scale subcritical water treatment of the four components (*l*-menthol, *l*-menthone, piperitone, and *l*-menthyl acetate) with an excellent throughput was successfully performed in batch mode. Each treatment was conducted on 10 µL of sample. The results of the treatments at 180–240 °C for 5–60 min are shown in Figure 1. Following GC analysis of the sample, the residual ratio was estimated by considering peak areas of the components. The results indicated that the residual ratio of *l*-menthol decreased to 89.3% at 180 °C after 60 min. In the same 60 min treatment, the ratio of *l*-menthol further decreased to 81.6% when the temperature was increased to 240 °C. As *l*-menthyl acetate maintained its residual ratio above 90% after treatment at 240 °C for 60 min, *l*-menthyl acetate was clearly more heat resistant compared to *l*-menthol. In the 60 min treatment, the residual ratio of *l*-menthone was 94.7% at 180 °C, decreasing to 82.2%, 71.3%, and 62.1% as the temperature increased to 200, 220, and 240 °C, respectively. That is, *l*-menthone was the most temperature sensitive among the four components. Piperitone exhibited moderate heat resistance. The residual ratio of piperitone was 89.9% at 180 °C in the 60 min treatment, and it decreased to 73.4% as the temperature was increased to 240 °C.

In our previous study, excellent yields of *l*-menthol and *l*-menthone could be obtained from Japanese mint leaves at 180 °C within 5 min. In this study, the residual ratios of *l*-menthol, *l*-menthone, *l*-menthyl acetate, and piperitone were 99.5%, 99.2%, 97.7%, and 99.3%, respectively, under the same treatment conditions (180 °C, 5 min). That is, subcritical water did not lead to significant loss of Japanese mint essential oil under optimal extraction conditions (180 °C, 5 min) [12].

### 2.2. Individual Component Treatments

**Figure 2:**
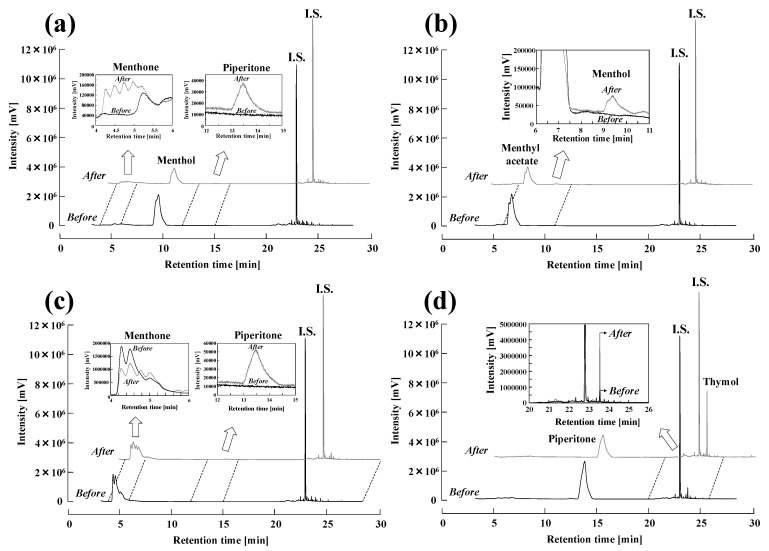
Individual treatments of the mint flavor components including (**a**) *l*-menthol, (**b**) *l*-menthyl acetate, (**c**) *l*-menthone, and (**d**) piperitone. Each of the subcritical water treatments was carried out at 240 °C for 60 min. Before the analysis of GC-MS, phenol (internal standard) was mixed with the treated sample and the final concentration of phenol was 1% (*w/v*).

Individual treatments of *l*-menthol, *l*-menthyl acetate, *l*-menthone, and piperitone were performed at 240 °C for 60 min, and the results before and after the treatments are shown as Figure 2a–d, respectively. As seen in Figure 2a, *l*-menthol exhibited a retention time of approximately 9.52 min, and the shoulder peak was ascribed to a stereoisomer of menthol, based on the GC-MS result. However, to simplify the discussion, the stereoisomer was not considered thereafter in this study. Following the treatment, the peak area of *l*-menthol decreased, while the peaks of menthone (Figure 2a, left inserted figure) and piperitone (Figure 2a, right inserted figure) appeared. *l*-Menthyl acetate gave rise to a peak with a retention time of approximately 6.64 min, as seen in Figure 2b. Analogous to *l*-menthol, *l*-menthyl acetate exhibited a shoulder peak close to the main peak, which was likewise attributed to a stereoisomer of menthyl acetate. The inset in Figure 2b indicated that the peak corresponding to *l*-menthol appeared after the treatment of *l*-menthyl acetate. The results suggest that the ester bond of *l*-menthyl acetate was hydrolyzed by subcritical water, and *l*-menthyl acetate was thereby converted into *l*-menthol. As the *l*-menthol peak was small, this indicated that it was likely immediately further degraded. The produced acetic acid was most probably responsible for accelerating the degradation by increasing ionic products under subcritical water conditions. Prior to treatment, *l*-menthone had a retention time of 4.50 min, while two shoulders appeared at 4.29 and 5.00 min, as evident in Figure 2c. After the treatment, one major peak (4.50 min) and three shoulders (4.27, 4.78, 5.00 min) were detected. According to the results of GC-MS, the three shoulders were all stereoisomers of *l*-menthone. The shoulders at 4.78 and 5.00 min became more prominent after the subcritical water treatment. These results suggested that the rearrangement reactions of menthone occurred under subcritical water conditions. In the right-hand inset of Figure 2c, a peak at 13.47 min is evident, which was concluded to arise from piperitone. That is, in addition to the rearrangement reactions, the conversion of *l*-menthone to piperitone also occurred under subcritical water conditions. In Figure 2d, the piperitone peak appeared at 13.61 min, and its peak area decreased following subcritical water treatment. A noted peak with a retention time of 23.54 min showed a large increase after the treatment, and was identified as thymol based on the mass spectral library of NIST 14 with a possibility of 60.2%. A peak at 24.56 min was identified as 1-isocyanonaphthalene with a possibility of 62.7%, which would be inaccurately assigned as a cyano derivative, that is, the origin of nitrogen was uncertain. Moreover, since all the other minority peaks was also never assigned with low possibility (below 30%) based on the mass spectral library. According to these results, piperitone was likely oxidized, and then converted to thymol under subcritical water conditions. 

### 2.3. Conversion of Piperitone to Thymol

**Figure 3:**
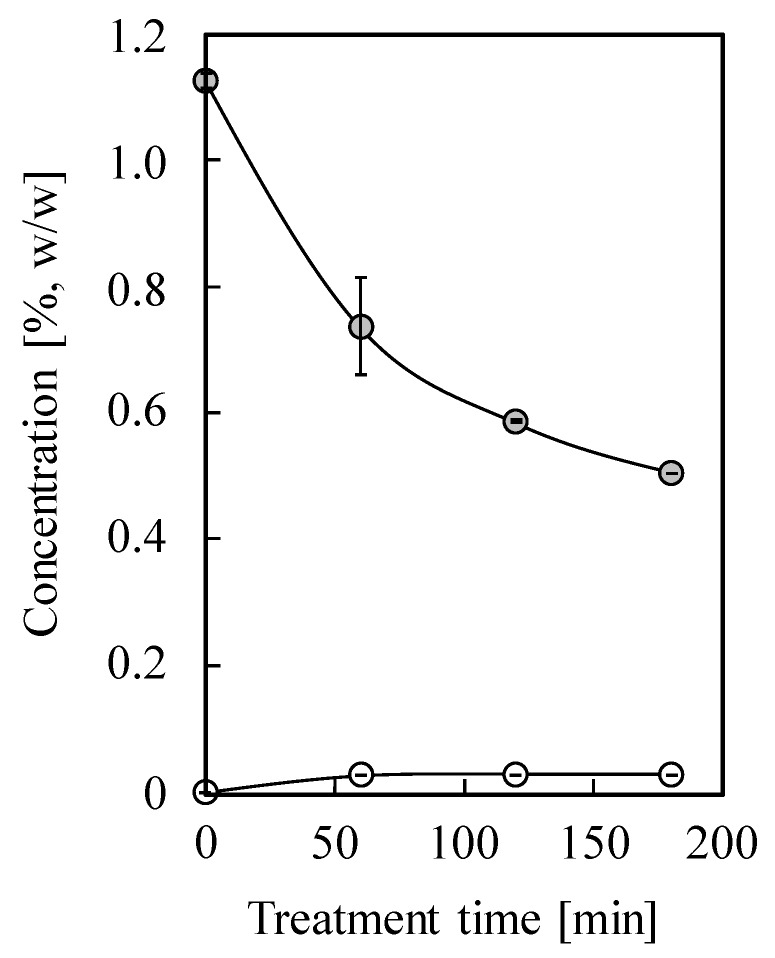
Conversion of piperitone (grey cycle) to thymol (white cycle). The subcritical water treatments were carried out at 240 °C for 0, 60, 120, and 180 min, respectively.

To further investigate the conversion of piperitone to thymol, a small-scale subcritical water treatment of piperitone was carried out at 240 °C for 60, 120, and 180 min, and the results are shown in Figure 3. The relative reference factors (RRFs) of piperitone and thymol to phenol were 1.02 ± 0.08 and 1.69 ± 0.08, respectively. The amount of piperitone decreased as the treatment time increased, decreasing to 0.51% (*w/w*) within 180 min. Approximately 0.03% (*w/w*) thymol was generated at 60 min; however, the concentration of thymol remained the same when the treatment time was extended to 120 min or to 180 min. It was therefore considered that thymol decomposed rapidly once it was generated from piperitone under subcritical water conditions at 240 °C. However, detailed elucidation of the mechanism and kinetics requires further investigation.

**Figure 4:**
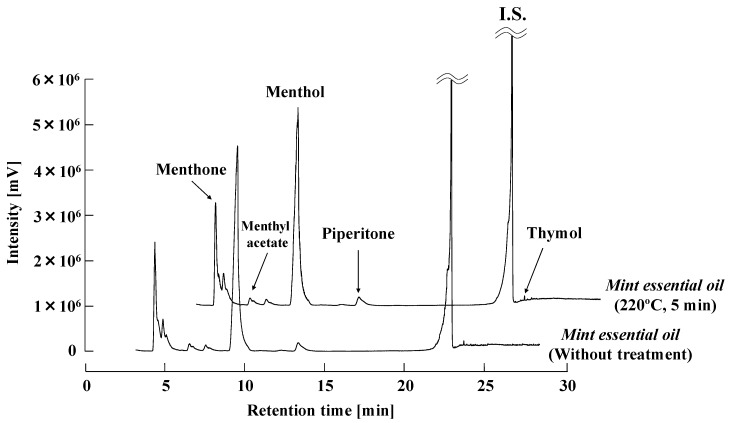
Thermal treatment of Japanese mint essential oil under subcritical water condition. The treatment was carried out at 220 °C for 5 min.

To investigate the reaction occurred in natural material, Japanese mint essential oil was treated at 220 °C for 5 min and the results compared with untreated sample are shown in Figure 4. The peak of menthol decreased approximately 7.5% based on the peak area (adjusted by internal standard), and this result was consistent with the result shown in Figure 3. The peak area of menthyl acetate decreased approximately 3.7%, which was lower than that of piperitone (9.8%). This result was also consistent with the result in Figure 3 for that menthyl acetate had higher thermal stability than piperitone under subcritical water condition. Since the peak of thymol increased approximately 13.1% after 5 min treatment at 220 °C, Japanese mint essential oil, as a natural material, was considered to have similar thermal decomposition characteristics to the standard reagents even the composition ratio of menthol in the oil was over 70% [12].

## 3. Materials and Methods 

### 3.1. Standard Reagents and Mint Essential Oil

Standard reagents, *l*-menthol (purity >99.0%), *l*-menthone (purity >85.0%), piperitone (mixture of enantiomers, purity >95.0%), and *l*-menthyl acetate (purity >98.0%) were purchased from TCI (Tokyo, Japan). Phenol was purchased from Sigma Aldrich, Japan (Tokyo, Japan). Thymol (purity > 98.0%), ethanol, and chloroform were procured from FUJIFILM Wako Pure Chemical Corporation (Osaka, Japan). All reagents used were reagent grade. Essential oil was obtained from dried Japanese mint (*Mentha arvensis* L. var. *piperascens* Malinv. cv) leaves by hydrodistillation extraction using an Herb oil maker (standard type for laboratory, TokyoSeisakushiyo, Tokyo) with a cylindrical tank (12.3 cm i.d. × 27 cm height). The extraction was carried out for two hours to treat 100 g dried mint leaves for one batch [12]. 

### 3.2. Small-Scale Subcritical Water Treatment of Mixture

**Figure 5:**
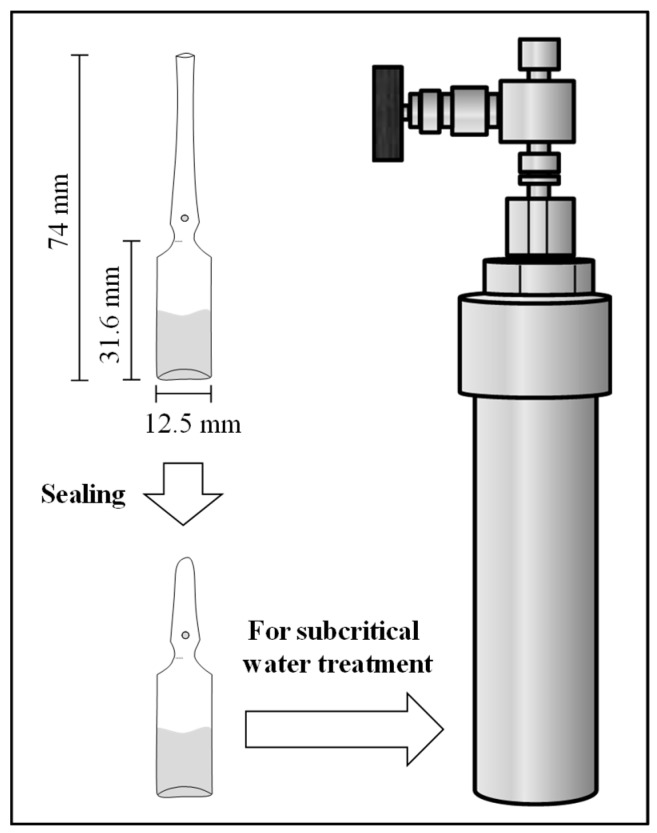
Illustration of small-scale subcritical water treatment.

Ampoule bottles (AP-2, Maruemu, Osaka) were used for small-scale subcritical water treatment. An oil mixture was made by mixing the same amount of each of the four components (*l*-menthol, *l*-menthone, piperitone, and *l*-menthyl acetate). An aliquot (10 µL) of the mixture and distilled water (1.0 mL) were added to an ampoule bottle (2 mL, AP-2, As One, Osaka) using a pipette, and the ampoule bottle was then sealed using a gas burner. The sealed ampoule bottle was placed in a pressure-resistant SUS-316 stainless steel vessel (TVS-N2 type, Taiatsu Techno, Osaka) (Figure 5). The internal volume of the vessel was 117 mL (3.0 cm i.d. × 16.6 cm height), and the maximum operation pressure and temperature of the vessel were 20 MPa and 260 °C, respectively. Distilled water (100 mL) was added to the vessel before the vessel was tightly fastened. A mantle heater (P5-2, Tokyo Garasu Kikai, Tokyo) equipped with a temperature regulator (TXN-700B, As One, Osaka) and a thermocouple (1.0 mm i.d. × 30.0 cm, K sheath 0–700 °C, As One, Osaka) were used for heating the vessel. To evaluate the thermal stability of the major components, the treatment temperatures were set at 180, 200, 220, and 240 °C, and the treatment times were set at 5, 10, 20, and 60 min. These treatment conditions were set based on the optimized treatment (180 °C, 5 min) proposed by our previous study [11,12]. Measurement of the treatment time commenced once the vessel had reached the desired temperature [13]. Phenol was used as an internal standard (I.S.) in gas chromatography (GC) analysis. Following treatment, the ampoule bottle was carefully opened and 1010 µL of 2.0% (*w/v*) phenol-acetone solution was added to the mixture. The well-mixed sample was further diluted 10-fold with acetone before GC analysis.

### 3.3. GC Analysis

A GC (Shimadzu, Kyoto) equipped with a TC-WAX capillary column (0.25 mm i.d. × 30.0 m; GL Science, Tokyo) was used for the quantification. Both the carrier gas and the make-up gas used were nitrogen gas. The total flow and purge flow were set 19.3 mL/min and 3 mL/min, respectively. The split ratio was set 20 to 1. Both the temperatures of the injector and FID detector were set to 250 °C. The program of oven temperature was set as follows: held at 50 °C for 3 min, increased to 110 °C at 15 °C/min, increased to 150 °C at 3 °C/min, increased to 200 °C at 15 °C/min, and held at 200 °C for 5 min. A 1.0 µL aliquot of each sample was injected into the GC in duplicate.

### 3.4. Individual Treatment of Mint Flavor Components

In addition to the mixture, the components (*l*-menthol, *l*-menthone, piperitone, and *l*-menthyl acetate) were individually treated by small-scale subcritical water treatment to investigate the possible mechanism of conversion and decomposition. Ten microliters of *l*-menthone, piperitone, or *l*-menthyl acetate, or 10 mg of *l*-menthol were individually added to an ampoule bottle (Figure 5) filled with 1.0 mL of distilled water. After sealing the bottle, the treatment was carried out at 240 °C for 60 min. After the treatment, the 2.0% (*w/v*) of phenol-acetone solution was added (1010 µL) to the sample. The mixed sample was further diluted 10-fold with acetone before gas chromatography-mass spectrometry (GC-MS) analysis.

### 3.5. GC-MS Analysis

Qualitative analysis of the treated sample was carried out by GC-MS (Agilent Technologies, Santa Clara, CA). As with the GC analysis, a TC-WAX capillary column (30 m × 0.25 mm i.d., GL Science, Tokyo, Japan) was used in GC-MS analysis. The carrier gas used was helium gas, and the split-less mode was set at 4.0 mL/min. The temperatures of both injector and quadrupole were set at 250 °C, and the temperature of the ion source was set at 170 °C. The oven temperature program was set as follows: held at 100 °C for 20 min, increased to 240 °C at 40 °C/min, and held at 240 °C for 5 min. A 1.0 µL aliquot of each sample was injected into the GC-MS in duplicate. The mass spectral library of NIST 14 was used for assigning the detected peaks.

### 3.6. Additional Treatments of Piperitone and Mint Essential Oil

In addition to the 60 min treatment described above, piperitone was further treated at 240 °C for 120 and 180 min, and GC analysis was conducted. The relative reference factor (RRF) between the samples and I.S. was evaluated. In order to confirm thermal stability of real mint essential oil, the Japanese mint essential oil described in *4.1.* Standard reagents and mint essential oil were used. The small-scale subcritical water treatment of the mint essential oil (5% aqueous solution, *v/v*) was carried out at 220 °C for 5 min. Both treated and untreated oil samples were mixed with the same volume of phenol-acetone solution (2.0%, *w/v*). The mixed samples were further diluted 10-fold with acetone before the GC-MS analysis. 

## 4. Conclusions

**Figure 6:**
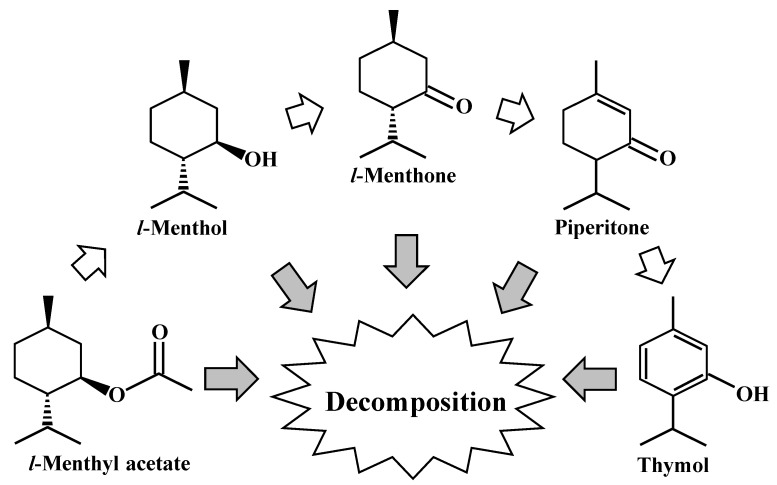
Schematic chart of conversion and hydrothermal decomposition of major components of mint essential oil during subcritical water treatment.

Small-scale subcritical water treatment was performed in order to evaluate the thermal stability of four major mint-essential-oil components; showed an excellent throughput. Under various temperature and duration treatments (180–240 °C, 5–60 min), the order of thermal resistance was *l*-menthyl acetate > *l*-menthol > piperitone > *l*-menthone. For the 5 min subcritical water treatment at 180 °C, the degradation ratios of the four components were all below 3%. Regarding the treatment of individual components (240 °C, 60 min), conversion and hydrothermal decomposition were observed. The high thermal stability of Japanese mint essential oil was also observed when the treatment was carried out at 220 °C for 5 min under subcritical water condition. As summarized in Figure 6, a portion of *l*-menthyl acetate was consequently converted into *l*-menthol, piperitone, and thymol. The decomposition of the four components and thymol had most likely occurred during or after the conversion. These results give insight into the degradation mechanism of mint essential oil components under subcritical water conditions, and provide the basis for optimizing extraction conditions of mint essential oils using subcritical water.
